# SPS-RCNN: Semantic-Guided Proposal Sampling for 3D Object Detection from LiDAR Point Clouds

**DOI:** 10.3390/s25041064

**Published:** 2025-02-11

**Authors:** Hengxin Xu, Lei Yang, Shengya Zhao, Shan Tao, Xinran Tian, Kun Liu

**Affiliations:** 1 College of Transportation, Shandong University of Science and Technology, Qingdao 266590, China; xuhx@sdust.edu.cn; 2 College of Ocean Science and Engineering, Shandong University of Science and Technology, Qingdao 266590, China; ts11ts06@163.com (S.T.); 202483190077@sdust.edu.cn (X.T.); 3 National Deep Sea Center, Qingdao 266237, China; zsy@ndsc.org.cn (S.Z.); liukun@ndsc.org.cn (K.L.)

**Keywords:** 3D object detection, light detection and ranging (lidar), point–voxel fusion, semantic-guided proposal sampling, cascade network

## Abstract

Three-dimensional object detection using LiDAR has attracted significant attention due to its resilience to lighting conditions and ability to capture detailed geometric information. However, existing methods still face challenges, such as a high proportion of background points in the sampled point set and limited accuracy in detecting distant objects. To address these issues, we propose semantic-guided proposal sampling-RCNN (SPS-RCNN), a multi-stage detection framework based on point–voxel fusion. The framework comprises three components: a voxel-based region proposal network (RPN), a keypoint sampling stream (KSS), and a progressive refinement network (PRN). In the KSS, we propose a novel semantic-guided proposal sampling (SPS) method, which increases the proportion of foreground points and enhances sensitivity to outliers through multilevel sampling that integrates proposal-based local sampling and semantic-guided global sampling. In the PRN, a cascade attention module (CAM) is employed to aggregate features from multiple subnets, progressively refining region proposals to improve detection accuracy for medium- and long-range objects. Comprehensive experiments on the widely used KITTI dataset demonstrate that SPS-RCNN improves detection accuracy and exhibits enhanced robustness across categories compared to the baseline.

## 1. Introduction

Three-dimensional object detection has rapidly become a research hotspot in both industry and academia, driven by the advancement of autonomous driving technology and smart cities [1,2]. Compared to purely vision-based methods, LiDAR-based 3D object detection offers significant advantages, such as robustness to lighting conditions and richer geometric information for 3D scene understanding. However, the sparsity and irregular distribution of point cloud data present substantial challenges for point cloud-based 3D object detection.

Researchers have proposed various single-stage [3,4,5,6] and two-stage [7,8,9,10,11,12] methods to address these challenges, yet they still face the following limitations: (1) a high proportion of background points in the sampled point set, and (2) lower accuracy in long-distance object detection. To mitigate limitation (1) and capture more foreground points, Shi et al. [13] proposed a keypoint sampling method that focuses on the area around the center of proposals and emphasizes local sampling. However, this method struggles to adequately cover foreground points due to its reliance on traditional farthest point sampling (FPS) and inherent proposal bias. Chen et al. [14] introduced global semantic information to identify keypoints related to foreground objects, which enhances global sampling. Nonetheless, this approach has limited effectiveness in accurately localizing outlier points. To address limitation (2) and enhance detection quality for distant objects, Fan et al. [15] and Cai et al. [16] adopted a cascade structure, replacing the single refinement network in traditional two-stage methods to improve learning capacity. However, the refinement process is affected by error propagation and accumulation across multiple downstream sub-stages.

To mitigate the above problems, this study considers that a balanced global–local point sampling approach can enhance detection quality, particularly for medium- and long-range vehicle detection. To obtain more representative keypoints, we propose a semantic-guided proposal sampling (SPS) method. This approach combines global semantic cues with local region information to ensure comprehensive coverage of foreground points across the entire point cloud. Additionally, we introduce a cascade attention module (CAM) [17], which aggregates features from different sub-stages to achieve more thorough proposal refinement.

In this work, we propose a multi-stage detection framework based on point–voxel fusion, named SPS-RCNN, for detecting high-quality 3D objects from LiDAR point clouds to achieve the above goal. The framework comprises three components: a voxel-based region proposal network (RPN), a keypoint sampling stream (KSS), and a progressive refinement network (PRN). In the RPN stage, the point cloud is voxelized using 3D sparse convolution, and 3D object proposals are generated by learning multi-scale features. In the KSS, the proposed SPS method combines local sampling based on proposals [13] with global sampling using semantic guidance [14]. This fusion increases the proportion of foreground points and improves sensitivity to outliers through global–local multilevel sampling. In the PRN stage, the accuracy of proposal refinement is progressively enhanced by establishing more effective connections across sub-networks using a CAM based on the multi-head self-attention (MHSA) mechanism.

In summary, our contributions are listed as follows:

1. In the downsampling stage, we propose semantic-guided proposal sampling (SPS), which integrates global and local sampling methods. For global sampling, distance values weighted by semantic information enhance the representativeness of keypoints, while local sampling refines FPS within the region around the proposal. Through global–local multilevel fusion sampling, SPS achieves a balanced and highly expressive keypoint distribution.
2. In the proposal refinement stage, we introduce CAM to aggregate multi-stage object features. This module enhances proposal refinement accuracy by progressively improving the proposal quality at each stage.
3. We conduct comprehensive experiments on the widely recognized KITTI dataset [18]. Compared to the PV-RCNN [19] benchmark, our approach achieves improved detection accuracy for the vehicle category and demonstrates strong robustness.

## 2. Related Work

### 2.1. Point-Based Sampling Algorithm

In point-based detection methods, most models [20,21,22] address the downsampling problem using classical farthest point sampling (FPS). To enhance point modeling capabilities, recent studies [13,14,23,24,25,26,27] have designed novel point sampling algorithms. Zhang et al. [23] developed the IA-SSD model with Ctr-aware sampling, fully leveraging the geometry of the bounding box. Shi et al. [13] advanced the sampling speed and the representativeness of sampled points through the sectorized proposal-centric (SPC) method. Additionally, some methods incorporate heuristic information into the sampling strategy. For instance, Feature-FPS (F-FPS) [24,25] considers the feature distances between points to improve the diversity of sampled features. To increase the sampling of foreground points and boost detection performance, Chen et al. [14] proposed semantics-guided farthest point sampling (S-FPS), leveraging direct semantic information. Subsequent studies [26,27] further improved S-FPS by integrating additional heuristic insights.

Previous point sampling methods either focus on ensuring global uniformity of the sampled point set or concentrate on local information to capture foreground features, but fail to effectively combine both. To address this limitation, we propose semantic-guided proposal sampling (SPS), which achieves comprehensive coverage of foreground points through a global–local fusion sampling approach.

### 2.2. Point/Voxel-Based 3D Object Detection

Current 3D detection methods [9,19,28,29,30,31,32,33,34] can be broadly categorized into point-based, voxel-based, and hybrid point–voxel methods, depending on how the point cloud is processed. Point-based methods detect 3D objects directly from the original point cloud. Dong et al. [28] developed a point-based detector that effectively mitigates proposal entanglement by incorporating contextual semantics. Wang et al. [29] extend point-based pipeline by grouping features to better capture geometric properties of objects. In contrast, voxel-based methods regularize the unordered 3D points into a structured grid format. An et al. [32] identified foreground voxels through saliency prediction (SP) to achieve relatively accurate 3D scene understanding under adverse weather conditions. Sun et al. [9] further improve detection accuracy by incorporating voxel attention and multiscale feature fusion. Furthermore, several studies [13,19,34] have integrated point-based and voxel-based approaches to enhance feature learning. Inspired by these works, we develop a point–voxel fusion detection framework that effectively leverages the advantages of both point cloud representations.

### 2.3. Multistage Network for Object Detection

Multi-stage (beyond two stages) approaches have been extensively demonstrated in 2D object detection [35,36] and are now being extended to 3D detection [16,17,37,38,39]. Cai et al. [16] proposed 3D Cascade RCNN, a generalized cascade paradigm for 3D object detection in complex scenes. To enhance detection quality, Wu et al. [17] introduced an attention mechanism into the cascade structure, proposing a novel cascade framework, CasA. Lu et al. [38] developed a hierarchical refinement network that adaptively selects samples at each sub-stage through dynamic sampling. This approach improves the model’s ability to learn from high-quality samples. Liu et al. [39] incorporated a cascade structure into multi-modal detection. The framework enhances point feature representation by leveraging image semantics within a bidirectional interactive fusion process. Unlike previous approaches, we design a progressive refinement network (PRN) within a multi-stage detector that employs a cascade attention module (CAM) to aggregate features from multiple sub-stages, progressively enhancing the quality of proposal refinement.

## 3. Methods

### 3.1. Overview

**Figure 1:**
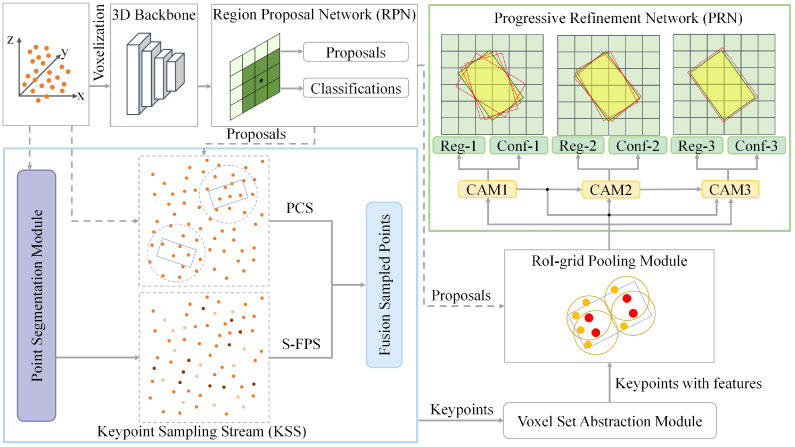
The overall architecture of our proposed SPS-RCNN, comprising a voxel-based region proposal network (RPN), keypoint sampling stream (KSS), and progressive refinement network (PRN).

The sparsity and irregularity of point clouds often result in low accuracy and high false positive rates in LiDAR-based detection. During the downsampling stage, the widely used farthest point sampling (FPS) frequently selects excessive background points that are irrelevant to the objects. While proposal-based sampling methods aim to improve the proportion of foreground points in the keypoint set through proposal localization, they inherently suffer from point omissions caused by proposal bias. To address these challenges, we propose a detection framework named semantic-guided proposal sampling-RCNN (SPS-RCNN), which enhances detection accuracy and reduces false detections through a novel hybrid global–local sampling method. As illustrated in Figure 1, the framework comprises three main components: a voxel-based region proposal network (RPN), keypoint sampling stream (KSS), and progressive refinement network (PRN).

To achieve accurate environment understanding and object localization, we employ a multi-source feature representation. This approach addresses the computational inefficiency and local feature extraction limitations of point-based methods while mitigating the quantization errors and detail loss inherent in voxel-based detectors. Specifically, point cloud features, voxel features, and bird-eye-view (BEV) features of keypoints are fused using the voxel set abstraction (VSA) module [19]. Point cloud features are extracted directly from the raw point cloud using PointNet++ [40], whereas voxel and BEV features are derived from the voxel-based RPN. In the 3D backbone, we adopt voxel CNN [19,41] to efficiently encode voxel features. The output tensor is compressed along the z-axis to generate BEV feature maps. Subsequently, the RPN generates object proposals and class predictions. To enhance contextual understanding, the VSA-fused multi-source features are further aggregated by the RoI-grid pooling module to produce RoI features. These RoI features, combined with RPN-generated proposals, are then fed into the PRN. The PRN outputs bounding box regressions and confidence predictions through the cascade attention module (CAM) [17]. By integrating cascade structures with attention mechanisms, CAM addresses the limitations of single refinement networks in learning fine-grained features and mitigates error propagation and accumulation in traditional multi-stage frameworks.

In the KSS, to increase the proportion of foreground points in the keypoint set and comprehensively capture object information, we propose a novel global–local semantic-guided proposal sampling (SPS) method. This approach integrates local sampling based on proposal-centric sampling (PCS) with global sampling using semantics-guided farthest point sampling (S-FPS) to achieve more complete and uniform coverage of foreground points. Note that the semantic information for global sampling is obtained through a point segmentation module.

### 3.2. Three-Dimensional Backbone for Proposal Generation

To accurately and efficiently convert the raw point cloud into sparse 3D feature volumes, we employ a voxel CNN with 3D sparse convolution [19,41] as the backbone for feature encoding and 3D proposal generation.

The backbone network first partitions the input point cloud into small voxels with a spatial resolution of L×W×H. For non-empty voxels, their features are computed as the average of the point-wise features of all inside points. The commonly used features include 3D coordinates and reflectance intensities. The backbone then progressively downsamples the point cloud into 1×, 2×, 4×, and 8× feature volumes using a series of 3×3×3 3D sparse convolution. After extracting features from the input point cloud, the encoded 8× downsampled 3D feature volumes are converted into 2D bird-view feature maps.

The next step involves generating high-quality 3D proposals in the RPN using the anchor-based approach [41,42]. Specifically, the $\frac{L}{8}\times \frac{W}{8}$ bird-view feature maps are obtained by compressing the 3D feature volumes along the z-axis. For each pixel in the feature maps, $2\times \frac{L}{8}\times \frac{W}{8}$ anchor boxes per class are generated, aligned with the average size of the objects in the respective class. Anchors are evaluated at orientations of $0^{\circ }$ and $90^{\circ }$, respectively, to account for different object directions.

### 3.3. Semantic-Guided Proposal Sampling

**Figure 2:**
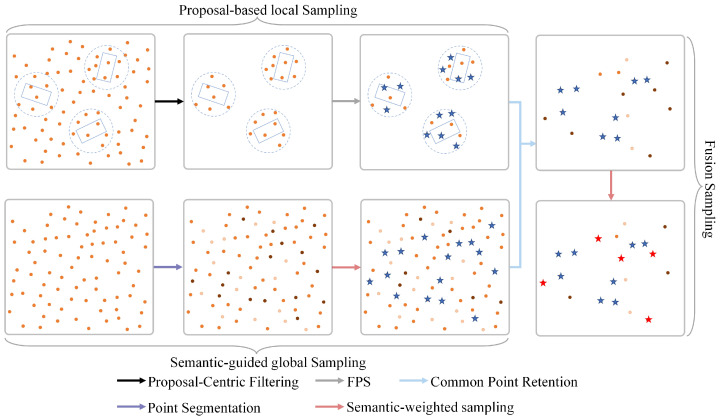
Schematic diagram of semantic-guided proposal sampling (SPS), comprising semantic-guided global sampling, proposal-based local sampling, and fusion sampling.

Considering that autopilot systems are primarily deployed in open scenarios, enhancing the detection quality of medium- and long-range objects is crucial. To address this, we propose semantic-guided proposal sampling (SPS), which captures more valuable spatial and positional information by acquiring a comprehensive set of foreground points. The structure of SPS is illustrated in Figure 2 and comprises three components: proposal-based local sampling, semantic-guided global sampling, and fusion sampling. In the Figure 2, dots denote the raw point cloud, stars indicate the sampled points, and arrows of different colors represent the intermediate processes.

In the proposal-based local sampling branch, to enhance keypoint sampling on medium- and long-range objects, inspired by PV-RCNN++ [13], we restrict the sampling region from the entire point cloud to discrete circular areas centered around each proposal. Within this localized region, we apply farthest point sampling (FPS) to ensure uniform coverage of all potential objects in the scene.

Due to proposal bias, the aforementioned proposal-based sampling is susceptible to the point omission problem. To overcome this limitation, and inspired by SASA [14], we adjust the sampling metric d (i.e., the distance to the already sampled point) of the vanilla FPS by incorporating the foreground semantic score p in semantic-guided global sampling branch, thereby improving the accuracy of foreground point selection. To obtain global semantic information, we embed a point segmentation module. This module consists of a two-layer MLP that classifies input points as either foreground or background. The foreground score p∈[0,1] for each point in the raw point cloud is calculated as

(1) $$p_{i}=\sigma \left(M\left(f_{i}\right)\right)$$

where $M\left(\cdot \right)$ denotes the point segmentation module, which maps the input point-wise feature $f_{i}$ to the foreground score $p_{i}$. σ(·) represents the sigmoid function.

For training the point segmentation module, foreground segmentation labels are directly derived from the box annotations. Points within any ground-truth 3D bounding boxes are treated as foreground points and the others as background ones. The total segmentation loss $L_{seg}$ is computed using the cross entropy (CE) loss function:

(2) $$L_{seg}=\frac{\lambda }{N}\cdot \sum_{i=1}^{N}\mathrm{CE}\left(p_{i},{\hat{p}}_{i}\right)$$

where $p_{i}$ and ${\hat{p}}_{i}$ represent the predicted foreground score and the ground-truth segmentation label (1 for points from foreground and 0 for ones from background) of the i-th point. N denotes the total number of input points, and λ represents the weight of the segmentation loss. As a result, the semantically weighted sampling metric ${\tilde{d}}_{i}$ in semantic-guided global sampling is formulated as follows:

(3) $${\tilde{d}}_{i}=d_{i}\cdot p_{i}^{\gamma }$$

where γ is a balancing factor that controls the importance of semantic information. When γ=0, the S-FPS simplifies to vanilla FPS, whereas if γ becomes very large, it approximates pure semantic sampling.

Finally, in fusion sampling, global and local sampled points are fused. Specifically, common points are retained, while distinct points are refined using semantic-guided sampling methods. The SPS approach based on fused sampling mitigates the limitations of semantic-guided sampling in handling distant outliers and addresses the issue of point omission caused by proposal bias in proposal-based methods. This enhances the algorithm’s capacity to capture a comprehensive and representative set of foreground points.

### 3.4. Progressive Refinement Network

Most current two-stage 3D object detectors rely on a single network to regress the 3D bounding box once during the proposal refinement stage. This approach often lacks detection accuracy, especially for medium- and long-range objects, and struggles to adapt to more complex scenarios. To address this issue, inspired by the work in [17], this paper introduces the cascade attention module (CAM).

**Figure 3:**
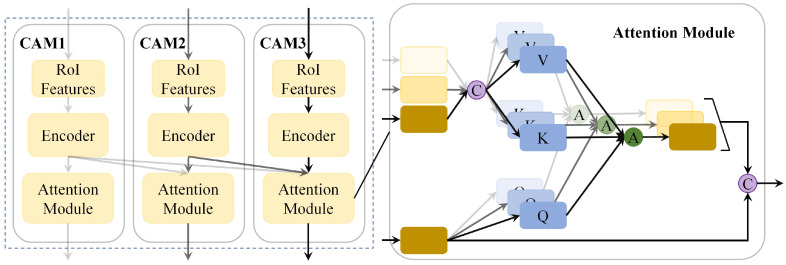
Illustration of the cascade attention modules (CAM) for progressively refining proposals through feature aggregation across multiple sub-stages.

Unlike existing multi-stage object detection networks [16], our CAM introduces attention mechanism [43] built upon the traditional cascade structure. CAM progressively refines the proposals by learning the importance of features at different stages and continuously aggregating them to generate the final detection. The specific structure is shown in Figure 3.

First, the keypoints with multi-source features are aggregated, and the RoI features $F^{j}$ are extracted using RoI-grid pooling [13,19]. Then, the output features from all previous stages are collected in the j-th stage, resulting in a new feature representation $F^{j}=\left[F^{0},F^{1},...,F^{j}\right]$ to provide more comprehensive information for the subsequent refinement process. After that, the query embedding $Q^{j}=F^{j}W_{q}^{j}$, key embedding $K^{j}=F^{j}W_{k}^{j}$, and value embedding $V^{j}=F^{j}W_{v}^{j}$ are generated through linear projections $W_{q}^{j}$, $W_{k}^{j}$, and $W_{v}^{j}$, respectively. Additionally, a multi-head design is incorporated, where the attentional value for the i-th head is computed by

(4) $${\hat{F}}_{i}^{j}=S\left[\frac{Q_{i}^{j}{\left(K_{i}^{j}\right)}^{T}}{\sqrt{D}}\right]V_{i}^{j}$$

where S[·] represents the softmax operation, and D denotes the feature dimension in multi-head attention. Since the features in the current stage significantly influence proposal refinement, we concatenate $F^{j}$ with multi-head attention features to ${\hat{F}}^{j}$, denoted as

(5) $${\hat{F}}^{j}=\mathrm{Concatenate}\left(F^{j},{\hat{F}}_{1}^{j},{\hat{F}}_{2}^{j},...,{\hat{F}}_{H}^{j}\right)$$

where H is the number of attention heads.

Note that in the first refinement stage, our module performs a self-attention operation. For subsequent stages, a cross-attention operation is employed. The features from the final stage are passed through two fully connected layers for confidence prediction and final bounding box regression. By utilizing the CAM, our model can more accurately estimate the quality of the proposals at each stage, thereby enhancing the precision of the proposal refinement process.

### 3.5. Training Losses

Our SPS-RCNN can be trained end-to-end using semantic segmentation loss $L_{seg}$, RPN loss $L_{rpn}$, and PRN loss $L_{prn}$. As previously mentioned, the semantic segmentation loss $L_{seg}$ is calculated by the CE loss function, as defined in Equation (2). The RPN stage loss $L_{rpn}$, as defined in SECOND [41], can be expressed as follows:

(6) $$L_{rpn}=L_{cls}+L_{loc}+L_{dir}$$

where $L_{cls}$, $L_{loc}$, and $L_{dir}$ denote the object classification loss, location regression loss, and direction regression loss, respectively. Specifically, $L_{cls}$ is calculated by the focal loss [44]. $L_{loc}$ is optimized with smooth-L1 loss for box regression, while $L_{dir}$ is computed by sine-error loss for angle regression.

The PRN loss $L_{prn}$ is the sum of the losses across all sub-refinement stages and is computed by

(7) $$L_{prn}=\frac{1}{N}\left[\sum_{i}\sum_{j}L_{conf}\left(\alpha_{i}^{j},{\hat{\alpha }}_{i}^{j}\right)+I\left({\mathrm{IoU}}_{i}^{j}>u^{j}\right)\sum_{i}\sum_{j}L_{\mathrm{reg}}\left(\delta_{i}^{j},{\hat{\delta }}_{i}^{j}\right)\right]$$

where $L_{conf}$ denotes the confidence loss, calculated using the binary cross-entropy loss [33], while $L_{\mathrm{reg}}$ represents the object box regression loss, computed by the smooth-L1 loss [45]. $\alpha_{i}^{j}$, ${\hat{\alpha }}_{i}^{j}$, $\delta_{i}^{j}$, and ${\hat{\delta }}_{i}^{j}$ indicate the confidence prediction, confidence target, residual prediction, and residual target, respectively, for the i-th proposal at the j-th refinement stage. $L_{\mathrm{reg}}$ are incurred only by proposals with ${\mathrm{IoU}}_{i}^{j}>u^{j}$, where ${\mathrm{IoU}}_{i}^{j}$ refers to the Intersection over Union (IoU) of the object box with the corresponding label, and N is the number of proposals.

The total loss of the algorithm is calculated by

(8) $$L_{total}=L_{seg}+L_{rpn}+L_{prn}$$

## 4. Experiments

### 4.1. Dataset and Evaluation Metrics

**KITTI Dataset:** The KITTI dataset is widely used for benchmarking 3D object detection methods in outdoor environments. In our study, we follow the standard protocol described in PV-RCNN [19], where the 7481 training samples (trainval-set) are split into a training set (3712 samples) and a validation set (3769 samples). The official test set consists of 7518 samples, for which the labels remain undisclosed. Consequently, we not only evaluate performance on the validation set, but also submit our results to the online server for test set evaluation. For the validation set, the models are trained using the train set, whereas for the test set evaluation, the models are trained on the entire trainval-set as in [19]. We utilize average precision (AP) as the evaluation metric, specifically with an IoU threshold of 0.7 for the car category. On 8 October 2019, the official AP calculation was updated from 11 recall points to 40 recall points. Accordingly, we report results using the AP40 metric for both the validation set and test set. To ensure consistency with earlier methods, we also report AP11 results on the validation set for comparative purposes.

### 4.2. Implementation Details

SPS-RCNN was trained from scratch in an end-to-end manner using the Adam optimizer, with an initial learning rate of 0.01 and a decay strategy based on the one-cycle learning rate schedule. The model was trained on two GTX 3090 GPUs with a batch size of 8, running for 80 epochs. The total training time on the KITTI validation set was approximately 18 h. During the proposal frame refinement phase, a random sample of 128 suggestion frames was selected, ensuring a balanced ratio of positive to negative samples (1:1). A suggestion frame was considered a positive sample for the refinement branch if it achieved at least a 0.55 3D Intersection over Union (IoU) with the ground truth frame; otherwise, it was classified as a negative sample. For model size and further evaluation, please refer to the “Inference Speed Analysis” section.

We employ OpenPCDet [46], an open-source toolbox tailored for 3D object detection tasks. OpenPCDet provides a highly modular and extensible framework for various LiDAR-based 3D detection methods, supporting a range of state-of-the-art algorithms, including PV-RCNN [19], SECOND [41], and PointRCNN [31]. Its streamlined design allows efficient model implementation, customization, and evaluation, making it particularly suitable for large-scale experiments and benchmarking on datasets like KITTI. The experimental environment and parameters of this paper are shown in Table 1.

During training, a 3D object detection data augmentation strategy was employed, which included random flipping along the yaw axis within the range of [−π/4, π/4] and global scaling factors randomly sampled from the range [0.95, 1.05]. Additionally, ground truth augmentation was applied by randomly pasting new ground truth objects from other scenes into the current training scene to simulate diverse object environments. In the inference stage, the top 100 regions of interest (RoI) candidate frames were first selected using non-maximum suppression (NMS) with an IoU threshold set at 0.7, meaning that detection frames were considered valid when their IoU exceeded 0.7. These proposed frames were then refined during the refinement stage by extracting keypoint features via set abstraction. Finally, another round of NMS with an IoU threshold of 0.1 was applied to eliminate redundant frames.

### 4.3. Evaluation on the KITTI Dataset

*(1) Validation Set:* We conducted quantitative experiments on the KITTI validation set, and Table 2 summarizes the performance comparison for the automotive category across three levels of difficulty: easy, medium, and hard. In the Table 2, the highest values are shown in **bold**, the second-highest are underlined, and improvements over the baseline are highlighted in red. SPS-RCNN outperforms the baseline PV-RCNN in the automotive class at all three difficulty levels, with improvements of 0.52%, 2.06%, and 0.43%, respectively, under the AP11 metric (average precision with 11 recall thresholds). Overall, our proposed SPS-RCNN achieves superior performance compared to existing methods. This improvement primarily stems from the semantic-guided proposal sampling strategy and the cascaded self-attention module. These design choices not only filter foreground points more effectively but also enhance the aggregation of proposal features across different refinement stages, leading to higher-quality 3D bounding boxes.

*(2) Test Set:* To further demonstrate the effectiveness of the hybrid sampling approach, we computed the average accuracy on the test set using 40 recalled positions from the official KITTI test server. Table 3 presents the performance of the algorithm on the KITTI test set. In the Table 3, the highest values are displayed in **bold**, the second-highest are underlined, and improvements over the baseline are highlighted in red. For the car category, SPS-RCNN improves the average precision (AP) by 0.54% and 0.39% compared to the PV-RCNN benchmark at the moderate and hard difficulty levels, respectively. For the cyclist category, it also shows significant improvements at the easy and hard difficulty levels, with increases of 2.06% and 2.03%, respectively. Furthermore, compared to state-of-the-art methods, SPS-RCNN achieves either optimal or near-optimal performance in both the moderate and hard difficulty levels for the car category, surpassing many classical LiDAR and camera fusion-based detection methods.

**Figure 4:**
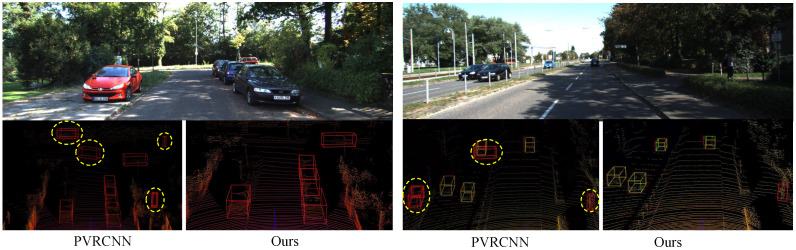
Visualization comparison between PV-RCNN and our method, demonstrating a significant reduction in vehicle misdetections.

**Figure 5:**
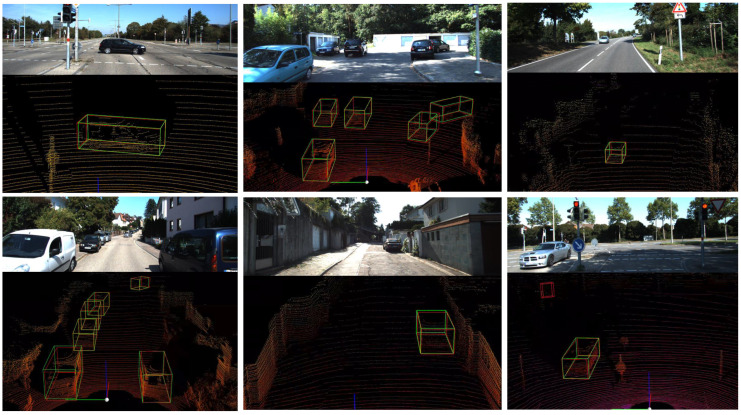
Three-dimensional visualization results of SPS-RCNN on the KITTI dataset, showing that the predicted 3D bounding boxes from our method almost perfectly align with the ground truth across various scenarios.

*(3) Visualized analysis:* We randomly selected traffic scenarios at various distances, and some visualization results of our algorithm on the KITTI dataset are shown in Figure 4 and Figure 5. The green bounding box represents the ground truth provided with the dataset, while the red bounding box represents the detection results from our algorithm. As shown in Figure 4, our proposed SPS-RCNN provides more accurate 3D predictions for vehicles compared to PV-RCNN. By highlighting the results for long-range object detection with yellow circles, we observe that the SPS-RCNN algorithm significantly reduces the missed detections present in PV-RCNN. Figure 5 illustrates the qualitative results of the improved SPS-RCNN in the KITTI scenario. The predicted 3D bounding boxes closely align with the ground truth for objects at varying distances and under different illumination conditions.

### 4.4. Ablation Study

***(1) Effectiveness of SPS Sampling Strategies:*** To validate the effectiveness of the hybrid sampling method, we compared the original PV-RCNN with three different sampling approaches: semantic-guided farthest point sampling (S-FPS), proposal-centric sampling (PCS), and semantic-guided proposal sampling (SPS), on the KITTI validation set. Table 4 summarizes the performance comparison for the AP40 metrics across various categories on the KITTI validation set. The experimental results show that compared to using only PCS, S-FPS significantly improves performance across different distance ranges for the car category, which supports the idea of using semantic information to weight distance values. Furthermore, compared to using either PCS or S-FPS alone, SPS achieves optimal results across all three difficulty levels (easy, moderate, and hard), further validating the effectiveness of our hybrid sampling approach. This method mitigates the problem of missing keypoints caused by candidate box bias in the keypoint sampling method within the proposal’s center region.

***(2) Effectiveness of different cascade stages:*** The original cascade structure can only capture the proposal features of the current stage while neglecting information from previous stages. To better learn the importance of features across different stages, we introduce the cascade attention module (CAM), which aggregates proposal features across stages and, in turn, produces higher-quality prediction frames. Table 5 shows the performance of SPS-RCNN when cascading from stage 1 to stage 3. The results demonstrate that increasing the number of cascade stages progressively improves detection accuracy for medium and hard vehicle categories. Since the best performance is achieved at stage 3, we adopt a three-stage cascade structure in this paper.

***(3) Effects of different components:*** To verify the effectiveness of each module in our algorithm, we conducted ablation experiments for all components, as shown in Table 6. All experiments were evaluated using the AP40 benchmark. Compared to the PV-RCNN benchmark, SPS-RCNN demonstrates a 1.18% improvement in the medium vehicle category. Specifically, by replacing the original FPS sampling with a hybrid sampling strategy guided by semantic proposals, SPS-RCNN achieves a 0.66% increase at the medium difficulty level for the car category, suggesting that the hybrid sampling strategy retains foreground information more effectively during the down-sampling process. Furthermore, in the proposal refinement stage, using the cascade attention module (CAM) instead of the original voxel RoI pooling results in an additional 0.52% improvement, highlighting that considering the quality of proposals across multiple stages can significantly enhance the accuracy of proposal refinement.

***(4) Inference speed analysis:*** To validate the impact of each component on model complexity and runtime, we progressively integrated them into the overall network, as summarized in Table 6. The vanilla PV-RCNN configuration, equipped solely with the FPS module, achieves the lowest parameter count and fastest runtime. However, it delivers suboptimal performance for 3D vehicle detection. Upon incorporating the SPS module, we observe a 0.66% improvement in 3D detection accuracy, with only a marginal increase in both model size and runtime, both of which remain within an acceptable range. Further integrating the CAM module achieves the optimal detection performance, with the parameter count increasing to 7.14 M and runtime rising to 62 ms. While the addition of multi-stage cascade strategies introduces some growth in model complexity and computational time, these trade-offs remain well within acceptable bounds. Such a balance between accuracy, model complexity, and runtime provides a valuable reference for deployment in real-world applications.

## 5. Conclusions

In this paper, we present SPS-RCNN, a multi-stage 3D object detector based on point–voxel fusion. This detector increases the proportion of sampled foreground points by integrating global semantics with local proposals during the downsampling process. Additionally, a cascade attention structure is utilized to refine region proposals, effectively addressing the challenge of low accuracy in distant object detection. Experimental results on the widely used KITTI dataset demonstrate that SPS-RCNN achieves significant improvements in detection quality compared to the PV-RCNN benchmark, particularly exhibiting strong performance and robustness in detecting medium- and long-range objects. Comparisons with state-of-the-art methods further validate the effectiveness of our proposed approach. We believe this design can be broadly applied to 3D object detection tasks in outdoor environments, including autonomous driving, robotics and autonomous navigation, augmented reality (AR) and virtual reality (VR). However, if the region proposal network (RPN) fails to generate proposals for small objects such as pedestrians, it may lead to missed detections. Future work will focus on enhancing detection accuracy for challenging small objects.

## Figures and Tables

**Table 1:** Experimental environment and parameters.

Parameter	Specification
CPU	Intel^®^ Xeon(R) Silver 4210R CPU
GPU	NVIDIA RTX 3090 × 2
GPU Memory	48 G
Operating System	Ubuntu 20.04
Deep Learning Framework	PyTorch 1.8.1
Python Version	3.8
CUDA Version	11.1
cuDNN Version	8.0
Code Editor	Visual Studio Code
Library	OpenPCDet

**Table 2:** Performance comparison for the vehicle category on the KITTI validation set using average precision at 11 recall positions (AP11).

Method	Reference	Car 3D AP (%)		
		Easy	Moderate	Hard
SECOND [41]	Sensors 2018	88.61	78.62	77.22
PointPillars [42]	CVPR 2019	86.62	76.06	68.91
PointRCNN [31]	CVPR 2019	88.88	78.63	77.38
3DSSD [24]	CVPR 2020	89.71	79.45	78.67
Part-A^2^ [47]	TPAMI 2020	89.47	79.47	78.54
TANet [48]	AAAI 2020	87.52	76.64	73.86
Voxel-RCNN [33]	AAAI 2021	89.41	84.52	78.93
CIA-SSD [49]	AAAI 2021	89.59	80.28	72.87
MFT-SSD [5]	RAL 2023	89.35	84.46	78.55
VFL3D [50]	TITS 2024	89.10	84.50	78.63
PV-RCNN [19]	CVPR 2020	89.35	83.90	78.70
SPS-RCNN	-	**89.87**	**85.96**	**79.13**
*Improvement (baseline)*		*+0.52*	*+2.06*	*+0.43*

**Table 3:** Performance comparison for the vehicle and cyclist categories on the KITTI test set, evaluated using average precision at 40 recall positions (AP40).

Method	Reference	Car 3D AP (%)			Cyc. 3D AP (%)		
		Easy	Mod.	Hard	Easy	Mod.	Hard
LIDAR + RGB							
MV3D [51]	CVPR 2017	74.97	63.63	54.00	-	-	-
F-PointNet [52]	CVPR 2018	82.19	69.79	60.59	72.27	56.12	49.01
UberATG-MMF [53]	CVPR 2019	88.40	77.43	70.22	-	-	-
EPNet [54]	ECCV 2020	89.81	79.28	74.59	-	-	-
3D-CVF [55]	ECCV 2020	89.20	80.05	73.11	-	-	-
LIDAR							
SECOND [41]	Sensors 2018	83.34	72.55	65.82	75.83	60.82	53.67
PointPillars [42]	CVPR 2019	82.58	74.31	68.99	77.10	58.65	51.92
PointRCNN [31]	CVPR 2019	86.96	75.64	70.70	74.96	58.82	52.53
3DSSD [24]	CVPR 2020	88.36	79.57	74.55	-	-	-
Point-GNN [56]	CVPR 2020	88.33	79.47	72.29	78.60	63.48	57.08
Part-A^2^ [47]	TPAMI 2020	87.81	78.49	73.51	79.17	63.52	56.93
TANet [48]	AAAI 2021	83.81	75.38	67.66	75.70	59.44	52.53
CIA-SSD [49]	AAAI 2021	89.59	80.28	72.87	-	-	-
Voxel-RCNN [33]	AAAI 2021	**90.90**	81.62	77.06	-	-	-
HVPR [57]	CVPR 2021	86.38	77.92	73.04	-	-	-
VIC-Net [58]	ICRA 2021	88.25	80.61	75.83	78.29	63.65	57.27
BADet [59]	PR 2022	89.28	81.61	76.58	-	-	-
SVGA-Net [60]	AAAI 2022	87.33	80.47	75.91	78.58	62.28	54.88
IA-SSD [23]	CVPR 2022	88.87	80.32	75.10	**80.78**	**66.01**	58.12
PV-RCNN [19]	CVPR 2020	90.25	81.43	76.82	78.60	63.71	57.65
SPS-RCNN	-	89.60	**81.97**	**77.21**	80.66	64.32	**59.68**
*Improvement (baseline)*		*−0.65*	*+0.54*	*+0.39*	*+2.06*	*+0.61*	*+2.03*

**Table 4:** Comparison of different sampling methods for vehicle classes on the KITTI validation set using average precision at 40 recall positions (AP40).

Methods	AP_3D_ (%)		
	Easy	Mod.	Hard
PV-RCNN	92.57	84.83	82.69
PV-RCNN + PCS	91.95	84.85	82.60
PV-RCNN + S-FPS	92.67	85.26	83.12
PV-RCNN + SPS	**92.79**	**85.49**	**83.19**

**Table 5:** Ablation study results on the KITTI validation set using different cascade stages, with the average precision (AP) for the car category is measured at 40 recall positions.

Stages	AP_3D_ (%)		
	Easy	Mod.	Hard
1	89.83	84.53	81.97
2	**92.12**	85.96	83.04
3	91.78	**86.01**	**83.26**

**Table 6:** Ablation study results of S-FPS and CAM on the KITTI validation set using average precision at 40 recall positions for the ‘car’ class.

FPS	SPS	CAM	AP_3D_ (%)	#Params	Runtime
✓			84.83	6.58 M	48 ms
	✓		85.49	6.72 M	55 ms
	✓	✓	86.01	7.14 M	62 ms

## Data Availability

Publicly available datasets were analyzed in this study. These data can be found here accessed on 23 August 2013: https://www.cvlibs.net/datasets/kitti.
